# *Curcuma longa* L. as a Therapeutic Agent in Intestinal Motility Disorders. 2: Safety Profile in Mouse

**DOI:** 10.1371/journal.pone.0080925

**Published:** 2013-11-18

**Authors:** Matteo Micucci, Rita Aldini, Monica Cevenini, Carolina Colliva, Silvia Spinozzi, Giulia Roda, Marco Montagnani, Cecilia Camborata, Luca Camarda, Alberto Chiarini, Giuseppe Mazzella, Roberta Budriesi

**Affiliations:** 1 Dipartimento di Farmacia e Biotecnologie, Università degli Studi di Bologna, Bologna, Italy; 2 Dipartimento di Scienze Mediche e Chirurgiche, Università degli Studi di Bologna, Bologna, Italy; 3 Dipartimento di Chimica “G. Ciamician”, Università degli Studi di Bologna, Bologna, Italy; Virginia Tech, United States of America

## Abstract

**Background:**

Curcuma extract exerts a myorelaxant effect on the mouse intestine. In view of a possible use of curcuma extract in motor functional disorders of the gastrointestinal tract, a safety profile study has been carried out in the mouse.

**Methods:**

Thirty mice were used to study the *in* *vitro* effect of curcuma on gallbladder, bladder, aorta and trachea smooth muscular layers and hearth inotropic and chronotropic activity. The myorelaxant effect on the intestine was also thoroughly investigated. Moreover, curcuma extract (200 mg/Kg/day) was orally administered to twenty mice over 28 days and serum liver and lipids parameters were evaluated. Serum, bile and liver bile acids qualitative and quantitative composition was were also studied.

**Results:**

In the intestine, curcuma extract appeared as a not competitive inhibitor through cholinergic, histaminergic and serotoninergic receptors and showed spasmolytic effect on K^+^ induced contraction at the level of L type calcium channels. No side effect was observed on bladder, aorta, trachea and heart when we used a dose that is effective on the intestine. An increase in gallbladder tone and contraction was observed. Serum liver and lipids parameters were normal, while a slight increase in serum and liver bile acids concentration and a decrease in bile were observed.

**Conclusions:**

Although these data are consistent with the safety of curcuma extract as far as its effect on the smooth muscular layers of different organs and on the heart, the mild cholestatic effect observed in absence of alteration of liver function tests must be further evaluated and the effective dose with minimal side effects considered.

## Introduction

In a previous paper [1] we have demonstrated that *Curcuma longa* L. extract exerts a myorelaxant effect on the ileum and colon of a mouse model of Sodium Dextran Sulphate induced colitis. The effect is not related to the well known antiinflammatory effect and it is more pronounced in the ileum in respect to the colon. The inhibitory activity of Curcuma towards basal and stimulated intestinal motility, associated with its spasmolytic and antispastic efficacy prompted us to propose the use of *Curcuma Longa* L. extract in the treatment of gastrointestinal tract functional disorders such as Irritable Bowel Syndrome (IBS), in addition to its use in the prevention of relapses and to maintain remission in Inflammatory Bowel Disease (IBD). Although IBS is a complex bio-psycho-social illness with a multifactorial etiology, involving, among others, diet and life style, altered bowel motility is a common feature, resulting in chronic abdominal dis-comfort, pain, associated with changes in bowel habits that compromise the quality of life. 

For centuries *Curcuma longa* L., the bright yellow spice, derived from the rhizome of *Curcuma longa* L., has been used in folk traditional medicine as a household remedy for a wide range of pathological conditions, such as arthritis, diarrhea and cancer [2,3]. Presently, a growing interest for herbal medicine has prompted a large amount of scientific investigations about the biological and pharmacological properties of curcuma extract main component, curcumin, which has been shown to exert several biological actions including anti-inflammatory [4-6], anti-infectious [7], antioxidant [8], antithrombotic [9], antiatherosclerotic [10], anticonvulsant [11] and anticancer [12-15] properties, cardio [16] and neuroprotective activities [17-20], in addition to improving memory [21], reducing aging [22], and determining benefits in psoriasis [23]. Moreover, curcumin seems to protect from metabolic syndrome [24] decreasing insulin resistance, obesity, hypertriglyceridemia, and hypertension [25] and to prevent the complications. Curcumin, due to its antioxidant and anti-inflammatory properties [8] is therefore a multifunction phytochemical [26] that can interact with multiple molecular targets, modulating cell growth, inflammation, and apoptosis signaling pathways [12]. The wide range of potential therapeutical clinical applications and the possible use in intestine functional motor disturbances prompted the present investigation to focus on the safety of this natural substance. In fact, IBS is very common in western countries [27] and IBS patients often complain of several concomitant associated pathological conditions, which may represent a controindication to its use. Therefore it is of main importance to evaluate the activity of this substance on other targets than the intestinal tract. Many animal [1,28] and clinical [3,6] reports demonstrating the therapeutic effect of curcumin are so far available and support its use [29-31] but few studies about curcumin toxicity, even at high doses, have been published [32,33]. Moreover a comprehensive and comparative investigation of the dose-related pharmacological activity of curcumin on different organs is not available in the same animal species. The potential use of curcumin as well as of the natural curcuma extract is severely hampered by poor water solubility of the active ingredients and short biological half-life of curcumin, resulting in poor intestinal absorption and low bioavailability irrespective of the administration rout [34-37]. Moreover lipid Curcuma extract carriers showed a significantly higher and earlier serum peak concentration and a greater AUC_0-∞_ compared with Curcuma extract [38]. The phytosomized extract significantly improves its oral bioavailability, and its tissue distribution in spleen, heart, liver, kidney, lung and brain [39].

Considering the potential use of *Curcuma longa* phytosomized extract in chronic conditions such as intestinal dysmotility disorders and in the prevention and maintenance of remission of IBD, requiring long time treatment [1], and due to the higher biodisponibility of this pharmaceutical form, it has seemed imperative a deep investigation following three main experimental approaches:

1. Intestine: to verify whether the *Curcuma longa* extract exerts any effect on other receptors systems or channels in the ileal tract and in the colon, the main intestinal segments involved in IBD, and to evaluate whether these effects, mainly on the cholinergic system, are also present in other gastrointestinal tract segments (stomach, jejunum and proximal colon);
2. Other systems :to evaluate whether other effects are present on the smooth muscular layers of the biliary tract, cardiovascular system, respiratory system, where it is recovered in high concentrations;
3. Liver, the main organ in lipid and xenobiotic metabolism,: to evaluate the parameters of liver function and serum lipids and, since administration of curcumin has been reported to induce a slight decrease in bile acid secretion (-12 %) in bile [40], to evaluate plasma, bile and liver bile acids qualitative and quantitative composition.

## Methods

### Animals

50 male Balb/c mice (8 weeks old, 25-30 g b.w.) (Charles Rivers Laboratories, Calco, Lc, Italy) were used. The animals were kept at constant light/dark cycling and constant room temperature of 22 °C. They were fed the usual commercial diet and tap water. The day before the experiment, food was withdrawn and water was allowed *ad libitum*. The animals were divided into 2 groups: a group (30 animals) was used as donors of the organs; a second (20 animals) group was administered over 28 days either 4RF21diet (Mucedola S.r.l., Milan, Italy) (10 animals) or 4RF21 complete food added with Curcuma (Indena Spa, Milan, Italy) extract at a final concentration of 1 g/kg. The delivery form of Curcuma used in the present study is a patented formulation of Curcumin (Free Curcumin; Curcuminglucuronide; Curcuminsulphate), a dietary phenolic, with soy lecithin. The two compounds form a non-covalent adduct in a 1:2 weight ratio, and two parts of microcrystalline cellulose are then added to improve formulation (www.phytosomes.info). The chemical preparation was proved to be 18-22% pure by HPLC total curcuminoids content [38]. Each mouse received 200 mg/kg per day, i.e. the human equivalent dose for mice [41]. 

### Experimental protocol

- *FunctionalStudiesonisolatedorgans*: immediately after the sacrifice, the organs of the donors animals were excised and immediately put in the appropriate buffer for functional studies (see below).
- *ChronicCurcumaadministration*: The day of the experiment, the animals fed with Curcumin (see above) and the controls were sacrificed by cervical dislocation: immediately after the sacrifice, blood was withdrawn by cardiac puncture in heparinated tubes, the gallbladder was removed, tied in the cystic duct and stored at-20°C until analysis. Similarly, the liver was excised and frozen at -20°C until analysis. Body weights, body weight gains/loss, feed consumption, and organ weights were monitored twice weekly. At the end of the experiment, terminal necropsy was performed for the gross and histopathological examination of the main organs.

#### Ethics statement

The work has been conducted according to the relevant National and International Guidelines. All experiments were conducted in conformity with the Public Health Service Policy on Humane Care and use of Laboratory Animals and approved by the Ethical Committee of the University of Bologna (PR 22.03.10). The animals were kept at constant temperature, light/dark cycle. Whenever a mouse gave signs of discomfort, the experiment was interrupted and the animal was switched to plain water and the usual commercial diet and excluded from the study.

### Isolated organs

For all assays male Balb/c mice (8 weeks old, 25-30 g b.w.) were used. The tissues were mounted in 15-ml organ bath containing appropriate solution. Ileum: Tyrode solution of the following composition (mM): NaCl, 145; KCl, 2.6; CaCl_2_·2H_2_O, 1.5; MgCl_2_·6H_2_O, 0.73; NaH_2_PO_4_·2H_2_O, 0.33; NaHCO_3_ 4.8; glucose 11.1; distal colon: Krebs solution of the following composition (mM): NaCl, 119; KCl, 4.5; CaCl_2_, 2.5; MgSO_4_·7H_2_O, 2.5; KH_2_PO_4_·2H_2_O, 1.2; NaHCO_3_ 25; glucose 11.1; Heart: Krebs solution of the following composition (mM): NaCl, 118; KCl, 4.7; CaCl_2_, 1.0; MgSO_4_·7H_2_O, 1.2; KH_2_PO_4_·2H_2_O, 1.2; NaHCO_3_ 25; glucose 11.1; Aorta: Krebs-Henseleit buffer solution of the following composition (mM): 118 NaCl, 4.8 KCl, 2.5 CaCl_2_, 1.2 MgSO_4_, 1.2 KH_2_PO_4_, 25 NaHCO_3_ and 11 glucose; trachea, gastric fundus, gallbladder and bladder: Krebs-Henseleit solution of the following composition (mM): NaCl, 118; KCl, 5.9; CaCl_2_, 2.5; MgSO_4_·7H_2_O, 1.2; Na_2_HPO_4_·2H_2_O, 1.0; NaHCO_3_ 25; glucose 10; thermostatically controlled at 37°C and buffered to pH 7.4 saturation with 95% O_2_-5% CO_2_ gas. After an equilibration period (60 - 90 min), the tissues were used to test curcuma extract (1 mg/ml) against specific receptor families (cholinergic, adrenergic, serotoninergic and CCK systems) and calcium channels, according to the tissue preparation method previously described [42]. In some tissues it was used a cumulative dosing regime for curcuma extract was used, and only one concentration-effect curve was obtained per preparation. The addition of the drug vehicle had no appreciable effect on different preparations.

- *Gastric fundus, gallbladder, ileum, distal colon and bladder assay preparations*. Each segment was removed, cleaned and mounted longitudinally under a resting tension of either 0.3g (stomach) or 0.5 g(gallbladder) or 1g (others). Tissues were allowed to equilibrate for 60 min during which time the bathing solution was changed every 10 min. Concentration–response curves to agonist were obtained at 30 min intervals, the first one being discarded and the second one used as control. A new concentration-response curve to agonist was obtained following incubation with the antagonist or Curcuma extract. Tension changes were recorded isometrically. In all cases, parallel experiments in which tissues did not receive any antagonist were run in order to check any variation in sensitivity. It was always verified that the EC_50_ values for the agonist in tissues receiving only the solvent were not significantly different (P > 0.05) from the control values. In all other cases experiments were discarded.
- *Heart assay preparations*. Atria. The experiments were carried out on spontaneously beating mice atria. An initial tension of 0.5 g was applied. Contractile activity was recorded isometrically by a force transducer (FT 0.3, Grass Instruments Corporation, Quincy, MA) using Power Lab software (AD-Instruments Pty., Ltd, Castle Hill, Australia). The right atrium frequency was also recorded. After beating for several minutes, a length-tension curve was obtained, and the muscle was stretched to the length at which 90% of maximal force was developed. A stabilization period of 45-60 min was allowed before the atria were challenged by various agents. During the equilibration period, the bath solution was changed every 15 min, and the threshold voltage was ascertained for the left atria. Atrial muscle preparation was used to examine the inotropic and chronotropic activities of the curcuma extract added to the preparation on a cumulative basis (in the range of 0.01-1 mg/mL) and the responses were recorded. During the generation of cumulative concentration-response curves, the next higher concentration of the extract was added only after the preparation reached a steady state.

Ventricles. The strips of left ventricle were driven at 1 Hz were used. The contractile activity was recorded isometrically by means of force transducer ( see above) The ventricular strips were stimulated by rectangular pulses of 0.6–0.8 ms duration and about 50% threshold voltage through two platinum contact electrodes in the lower holding clamp (Grass S88 Stimulator). The ventricles preparations were then processed as the atria.

- *Aorta assay preparations*. The thoracic aorta was removed immediately proximal to the heart, cleaned and placed in Krebs-Henseleit buffer solution. It was subjected to a resting force of 0.3 g and washed every 20 min with fresh PSS for 90 min. After the equilibration period; the contraction was effected by washing in PSS containing 80 mM KCl (equimolar substitution of K^+^ for Na^+^). After the (isometric) contraction reached a plateau (about 45 min) curcuma extract (0.001, 0.005, 0.01, 0.05, 0.1, 0.5, 1 mg/ml) was cumulatively added to the bath allowing for any relaxation to obtain an equilibrated level of force.
- *Trachea assay preparation*. Rings of trachea (3 mm) were mounted and placed under 1g resting tension. Tissues were allowed to equilibrated for 60 min, during which time the organ bath fluid was replaced at 30 min intervals.
- *Spontaneous contraction*. For gallbladder, gastric fundus, trachea and bladder the tracing graphs of spontaneous phasic contractions were continuously recorded. At the end of experiments, the amplitude of spontaneous phasic contraction (the difference between the basal level and the peak value was measured). The amplitude of the peach (milligrams) and the frequency (cycle per minute) as measured.

#### Statistical Analysis

Data were analyzed by Students t-test. The potency of the drugs defined as EC_50_, EC_30_ and IC_50_ was evaluated from log concentration–response curves (Probit analysis by Litchfield and Wilcoxon, n = 6–8) in the appropriate pharmacological preparations. All data are presented as mean ± SEM [43,44]. The dissociation constants of functional antagonism of curcuma extract *vs* histamine induced contraction, was calculated as previously described [42]. A pharmacological computer program [43] was used to analyze data. It was always verified that EC_50_ values were not significantly different (*P* > 0.05) from control values for the agonist in tissues receiving only the solvent. In other cases ex Figures were created using GraphPad software [45]. Statistical analysis was performed by using a two-tailed Student’s *t* test for serum parameters.

### Chronic Curcuma administration

- *Serum liver enzymes, lipids and glucose levels*

Alanine transaminases (AST) and aspartate transaminase (ALT), Triglyceride, HDL-cholesterol, total cholesterol and glucose concentrations were measured using Dimension RxL Max system (Siemens Healthcare Diagnostics, Newark, DE, USA), following manufacturer’s instructions. For each mouse a serum specimen volume of 100 µl was tested for each mouse.:

- *Bile acid analysis.*

Total bile acids in plasma, liver and bile have been determined by HPLC-ES-MS/MS [46]. Briefly HPLC was performed using a 2695 Alliance system (Waters, Milford, MA) separation module coupled with autosampler. The analytical column was a Luna Phenyl-Hexyl (150x2.0mm i.d., 4 µm particle size), protected by a Security Guard ODS 4 x 2.0 mm i.d. guard column, both supplied from Phenomenex. 

The sample preparation (extraction method) is different from a matrix to another.

Liver samples: each mouse liver sample was weighed and 0.60 g ± 0.2 g was taken from different points of the sample; 2 ml phosphate buffer (0.005 M, pH 7.2) was added and the mixture was homogenized ,washed with methanol (3 × 1 ml). The mixture was sonicated for 5 minutes, vortexed for 2 minutes, heated to 37°C for 20 minutes, and centrifuged at 4000 rpm for 15 minutes. 1ml of the supernatant (5 ml total) was dried under vacuum and resuspended with 2 ml sodium hydroxide (0.1 N). The solution was sonicated for 10 minutes, heated to 64°C for 30 minutes, and SPE was carried out on C18 extraction cartridges. The SPE cartridge was conditioned with 5 ml methanol and 5 ml water prior to sample loading. The liver sample extract was loaded onto the conditioned cartridge and washed with 10 ml water. The cartridge was then eluted with 5 ml of methanol. The eluate was dried under vacuum and reconstituted with 500 µl mobile phase solution; 5 µl were injected into the HPLC-ESI/MS instrument.

Bile samples: mouse bile samples were brought to room temperature, briefly stirred, and 1: 10000 with mobile phase. Final solution was transferred in autosampler vials, and 5 µl were injected into the HPLC-ESI/MS instrument.

Plasma samples: plasma samples (50 µl) were diluted 1:6 (v/v) with NaOH 0.1N and heated to 64°C for 30 minutes. The solid phase extraction (SPE) cartridge was conditioned with 5 ml methanol and 5 ml of water prior to sample loading. Plasma samples were loaded into the conditioned cartridge and then washed with 10 ml water. The cartridge was then eluted with 5 ml methanol and the eluate was collected, then dried under vacuum and reconstituted with 200 µl mobile phase and 5 µl were injected into the HPLC-ESI-MS/MS instrument.

Plasma free (for calibration curve): charcoal was rinsed several times, then dried. 50 mg of charcoal was put in every 1 ml of plasma and shaken. After stirring at +4 °C overnight, the plasma was centrifuged at 3000 rpm for 5 minutes, then filtered through Millipore µm 0.22 and stored at -20 ° C. 

Bile acids from different media were separated in elution gradient mode using 15 mM ammonium acetate buffer (pH = 8.00) as mobile phase A and acetonitrile: methanol =75:25 v/v as mobile phase B. Mobile phase B was increased from 30% to 45% in 10 min, then to 70% in 10 min, and finally brought to 100% in 1 min and held constant for 3 min. Injected sample volume was 5 µL. Flow rate was 150 µL/min and the column was maintained at 45 °C. The HPLC is combined with a triple quadrupole mass spectrometer with an Electrospray source operating in negative ionization (HPLC-ESI-MS/MS) using a Quattro-LC (Micromass) triple quadruple mass spectrometer operating in Multiple Reaction Monitoring (MRM) acquisition mode. MassLynx software version 4.0 was used for data acquisition and processing. Nitrogen was used as nebulizer gas at 100Lh.1 flow rate and as desolvation gas at 610Lh.1. Ion source block and desolvation temperatures were set at 120 °C and 180 °C, respectively. Capillary voltage was 3.1 kV and cone voltage was 60 V. Full-scan mass spectra and product ion spectra of each bile acids are obtained in the optimized mass spectrometry conditions. Chromatograms were acquired using mass spectrometer in multiple reaction monitoring (MRM) mode Collision energies was performed for each bile acids. The Micromass Mass-Lynx version 4.0 software was employed for instrument control, data acquisition, and processing. Calibration samples were obtained in the 0.05 to 20 μmol/L concentration range prepared in plasma free (same preparation used for the samples) and only in mobile phase. Linear calibration curve parameters were obtained from the plot of the analyte peak area versus Internal standards concentration using a least squares regression analysis (weight = 1/x). Correlation coefficients were higher than 0.991.

### Materials

Carbachol, atropine and all chemicals were obtained from Sigma (St. Louis, MO, USA). All solvents were of high purity and were used without further purification. Water LiChrosolv® for HPLC, MERCK was used. The standards of the different endogenous bile acids were obtained from Sigma Aldrich (St. Louis, USA); 6α-Ethil-chenodeoxicolic acid (6-ECDCA), its tauro- and glyco-conjugates were supplied by Prof. Roberto Pellicciari, University of Perugia, Italy and were highly pure (> 99 %) as documented by HPLC-ES-MS-MS analysis.

## Results

### Functional studies on isolated organs

In a previous investigation [1] we have evaluated the activity of *Curcuma longa Linn* extract in the smooth muscle of mouse ileum and distal colon: in these intestinal segments curcuma extract both decreased the resting tone and amplitude of basal contractions and inhibited the maximum response to Carbachol in a noncompetitive manner [1]. This relaxation on mice ileum and colon, already reported in the Guinea Pig by Itthipanichpong and colleagues [47] was reversed after 30 min tissue washing. The IC_50_ value for Curcuma extract was 0.031 mg/ml in ileum and 0.047 mg/ml in distal colon respectively[1]. In both cases, the agonist activity was comparable.

- *Ileum and distal colon*

*Effect on histamine induced contraction (ileum*)*.* Curcuma significantly affected histamine mediated tissue contraction. *Curcuma Longa Linn* extract (1mg/ml) reduces by 100% histamine induced contraction. The 100% inhibition is occurs already at a concentration of 0.05 mg/ml. Potency is reported in Table 1. This finding is consistent with the potent antiinflammatory effect of curcuma and its therapeutic activity [1]. 

**Table 1:** Antagonist affinity, expressed as IC_50_ values, in mice ileum smooth muscle segments.

	**Curcuma Extract**	
	**IC_50_** ^a^	**95% conf lim**
**Ileum**	0.022	0.012–0.031

^a^ IC_50_ is expressed as mg/ ml conc. and calculated from concentration-response curves. (Probit analysis by Litchfield and Wilcoxon with *n* = 6–7) [43]

**Figure 1:**
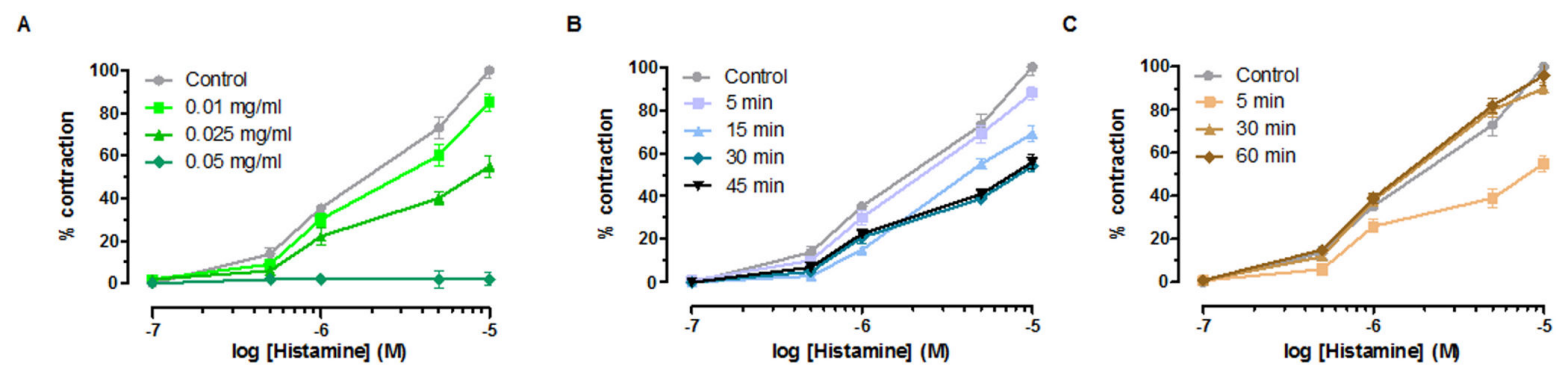
Effect of curcuma on histamine-induced contraction in isolated mice ileum. (A) Cumulative concentration-response curves were obtained before and after exposure to curcuma extract for 30 minutes. Data are mean ± SEM values (*n* = 5-6). (B) Time course of curcuma extract effect on histamine-induced contraction in isolated mice ileum (100%). Cumulative concentration-response curves were obtained before and after exposure to Curcuma extract (0.025 mg/ml) for 5, 15, 30, and 45 minutes. Data are mean ± SEM values (*n* = 4-7). (C) Time course of effect of curcuma-extract (0.025 mg/ml) on histamine-induced contraction in isolated mice ileum. Cumulative concentration-response curves were obtained before and after exposure to curcuma extract (0.025 mg/ml) and following washing for 5, 30, and 60 minutes. Data are mean ± SEM values (*n* = 3-5). Error bars are not shown where they are covered by the point itself.

The biological activity of curcuma extract against histamine-induced contraction was studied in the isolated mice ileum. As shown in Figure lA, curcuma extract reduced the maximum response to histamine in a concentration-dependent manner showing a noncompetitive antagonist mechanism. The maximum effect was reached within 30-minutes incubation at a concentration of 0.025 mg/ml (Figure 1B). The dose-response curve obtained with histamine after 45-minutes incubation (curcuma extract concentration: 0.025 mg/ml) did not differ from the curve obtained after are reversible, we studied the concentration-response curves to histamine after exposure to curcuma extract (0.025 mg/ml) at different washout times. As shown in Figure lC, the response to histamine was completely recovered after 60 minutes tissue washout.

**Figure 2:**
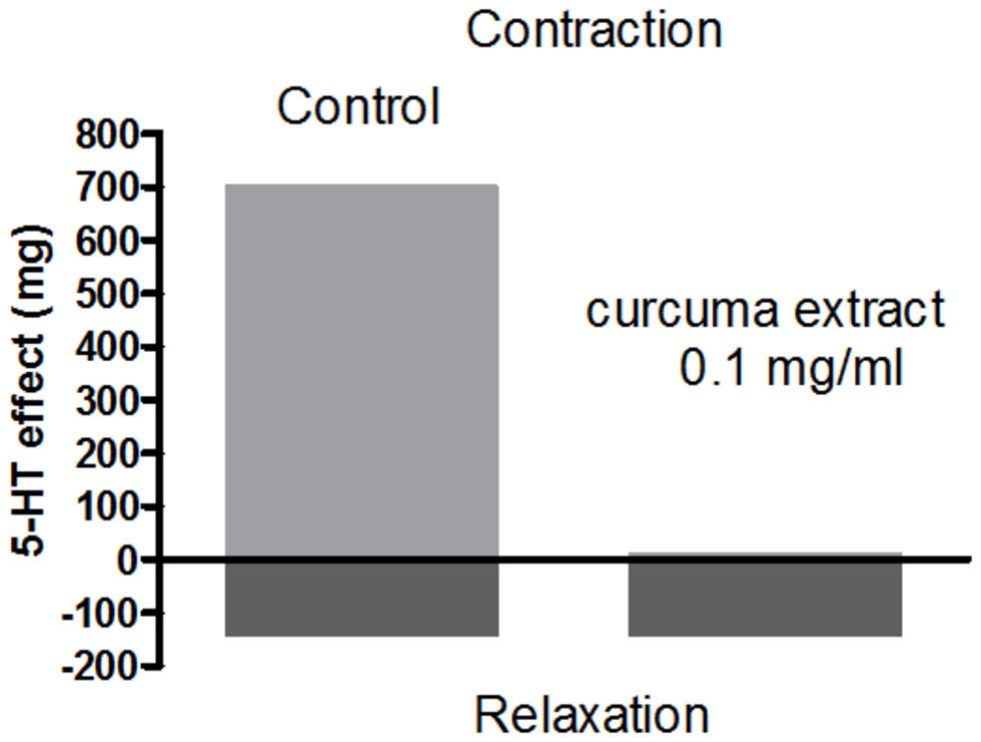
Effect of curcuma extract on 5-HT biphasic activity (contraction and then relaxation) in mouse distal colon. Mouse distal colon smooth muscle has been exposed to 5-HT (50 µM) before (control) and after exposure to 0.1 mg/ml curcuma extract for 30 min.. Data are mean ± SEM values (*n* = 3-5). Error bars are not shown where they are covered by the point itself.

*Effect on 5-HT activity (distal colon*)*.* On the distal colon, 5-HT exerts two contrasting actions: firstly it induces contraction, secondly it determines relaxation. The first activity is mediated by release of acetylcholine from the intramural parasympathetic ganglion cell cholinergic receptors, while relaxation is achieved via a direct effect on serotoninergic receptors. Although pre-incubation with curcuma (0.1 mg/ml) abolishes the contraction, it has no effect on the relaxation induced by serotonine (50 µM) (Figure 2). At a double dose is twice than the one inducing 50 % effect on CCh induced contraction on distal colon [1]. 

**Figure 3:**
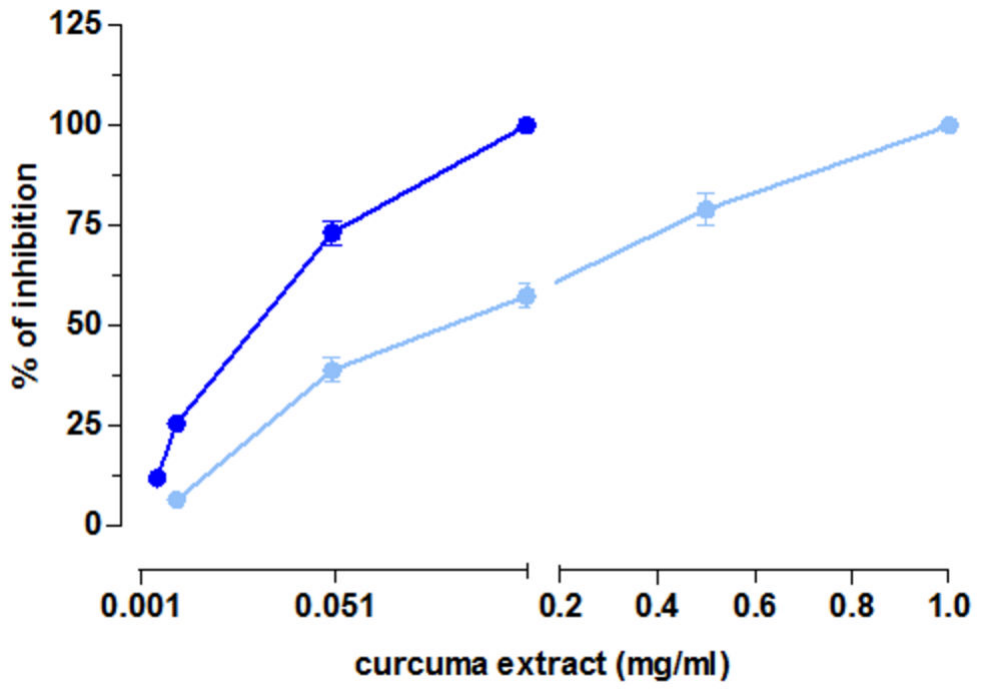
Spasmolitic effect of curcuma extract on K^+^ (80 mM) induced contraction on isolated ileum (dark blu) and on isolated distal colon (light blu). Dose-response curves of curcuma extract. Data are means ± SEM values (*n* = 3-5). Error bars are not shown where they are covered by the point itself.

*Effect on K* ^*+*^ *(80 mM*)* depolarized ileum and distal colon.* We have also evaluated the effect of Curcuma extract (1mg/ml) inhibits by 100 % K^+^ (80 mM) induced contraction in the ileum and reduces by 84 % K^+^ (80 mM) induced contraction in the distal colon (Figure 3A). Therefore, the direct activity towards the intestinal smooth muscle is exerted not only through the cholinergic receptors, but also at the level of L-type Calcium Channels (LTCC) [48]. The maximal activity is achieved at a dose 10 times higher in the colon than in the ileum (the intrinsic activity is 79 ± 0.9 at 0.5 mg/ml curcuma extract concentration in the colon and 73 ± 0.2 at 0.05 mg/ml in the ileum) (Table 2 and Figure 3). 100% contraction inhibition is obtained at 1mg/ml and 0.1 mg/ml in the distal colon and ileum respectively. We have also evaluated the potency of the inhibition: the effect is stronger in the ileum than in the colon, as theIC_50_ is 4.24 times less in the ileum than in the colon (Table 2).

**Table 2:** Calcium antagonist activities on mouse K^+^ (80 mM) depolarized ileum and distal colon.

	**Ileum**			**Distal Colon**		
	**Activity^a^ (M ± S.E.M.)**	**IC_50_^b^ (μM)**	**95% conf lim (x10^-6^)**	**Activity^c^ (M ± S.E.M.)**	**IC_50_^b^ (μM)**	**95% conf lim (x10^-6^)**
**CurcumaExtract**	73 ± 0.2	0.021	0.0092–0.035	79 ± 0.9	0.089	0.066–012

^a^ Percent inhibition of calcium-induced contraction on K^+^-depolarized (80 mM) mouse ileum longitudinal smooth muscle (0.05 mg/ml). Curcuma extract (0.1 mg/ml) inhibits contraction by 100%. ^b^ Calculated from concentration-response curves (Probit analysis by Litchfield and Wilcoxon [43], with *n* = 6-7). When the maximum effect was < 50%, the IC_50_ values were not calculated. ^c^ Percent inhibition of calcium-induced contraction on K^+^-depolarized (80 mM) mouse distal colon (0.5 mg/ml). curcuma extract (1 mg/ml) inhibits contraction by 100%.

- *Gallbladder*

**Figure 4:**
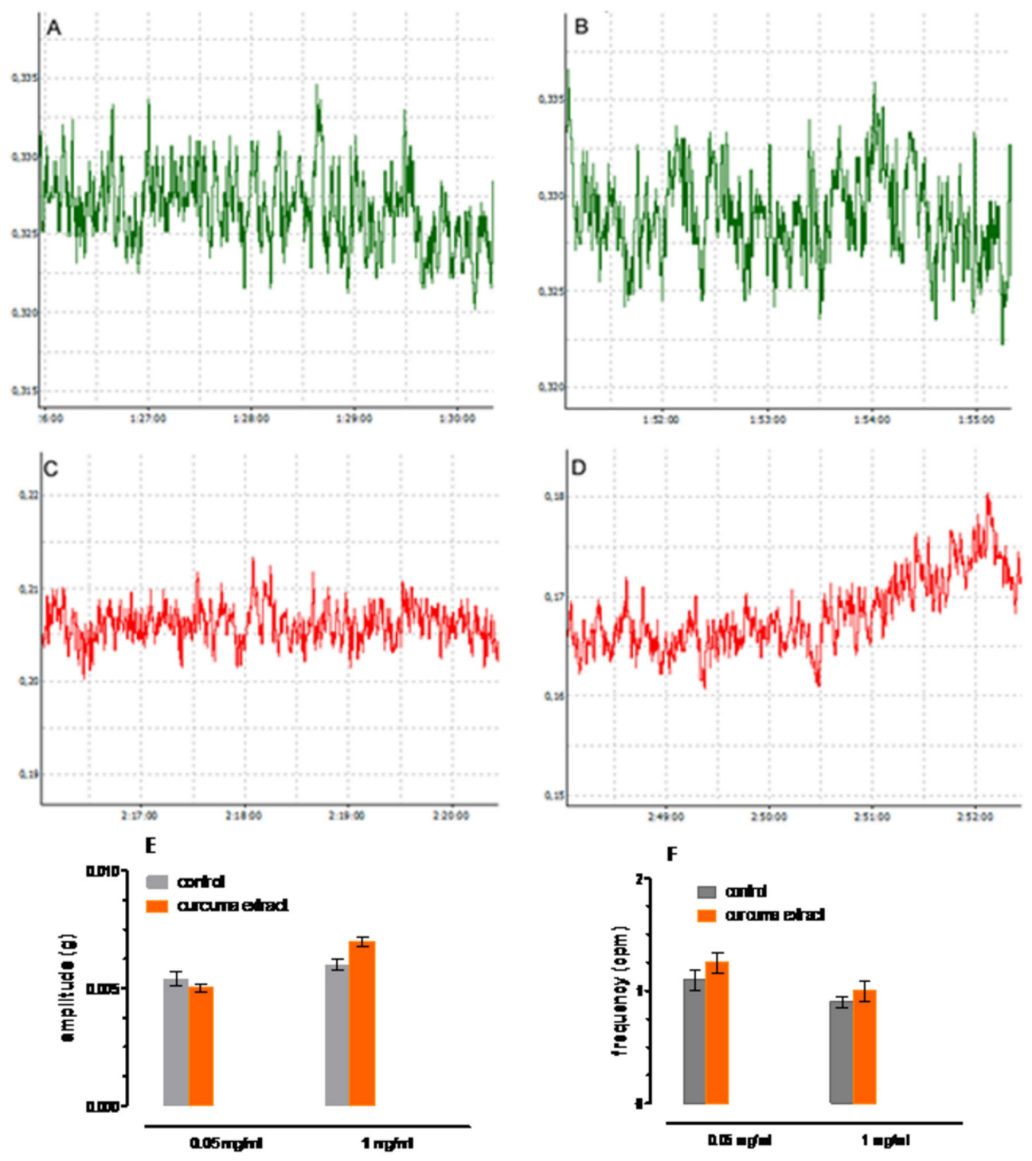
Gallbladder basal contractile activity. *Upper panels*: Representative traces of the spontaneous contractile activity (taken from Power Lab files) showing the basal contractile activity of the gallbladder smooth muscle before (A and C) and after the administration of Curcuma extract (0.05 mg/ ml (B) and 1mg/ml (D). X axis (g) and Y axis (h). *Lower* *panel*: amplitude (E) and frequency (F) of spontaneous phasic contractions. Data are mean ± SEM values (*n* = 6-7) (*P* > 0.05 curcuma vs controls).

The spontaneous contractile activities of isolated gallbladder smooth muscle preparations were not very regular: most of them showed tonic contraction, while few strips presented spontaneous phasic contractions. Curcuma (0.05 mg/ml) did not modify either the resting tone and the amplitude of spontaneous contractions (Figure 4B *vs* Figure 4A) or the phasic contractile activities. Increasing the concentrations of Curcuma to 1mg/ml did not produce any effect (Figure 4D *vs* Figure 4C). In particular the frequencies of spontaneous phasic contractions of muscle strips from control and after addition of curcuma extract (0.05 mg/ml) were not significantly different (n = 6, *P* > 0.05). The same holds for curcuma extract 0.1 mg/ml (n = 7, *P* > 0.05) (Figure 4E). The amplitudes of spontaneous phasic contractions of control muscle strips were not significantly different from those treated with curcuma extract 0.05 mg/ml (n = 7, *P* > 0.05) and with curcuma extract 0.1 mg/ml (n = 7, *P* > 0.05) (Figure 4F).

At the concentration of 0.05 mg/ml, which is the concentration active on the ileum and colon, the induced contraction is 30 % of that induced by Carbachol as reference. At the concentration of 1mg/ml, the natural extract increased by 255 % gallbladder contraction compared to Carbachol. In both cases, the effect was reversed by washing at both the concentrations. Since curcuma extract addition does not interfere with Carbachol induced contraction, it can be inferred that it probably does not compete with cholinergic receptors.

- *Left & right atria and ventricles*

As regards the cardiovascular system, we tested the effect of curcuma extract at a concentration of 1mg/ml, which is a concentration 300-400 times higher than the one inducing 50 % competitive reversible inhibition of Carbachol induced contraction in mouse ileum and distal colon respectively [1]. Curcuma decreased inotropy and chronotropy by 27 % and 39 % respectively. Since it was possible that these minimal effects could be due to agonism effect towards the cholinergic or adenosine systems, we have investigated these pathways. The effect of curcuma extract (1 mg/ml) was the same both in presence and absence of either atropine (1 μM) a known cholinergic antagonist and DPCPX (1 µM), a known adenosinic antagonist, which did not modify the inotropic and chronotropic negative effects of curcuma extract. Curcuma extract effects on cardiac parameters are reported in Table 3

**Table 3:** Curcuma extract effects on cardiac parameters.

	**Left & Right atrium**		**Ventriculum**
	**Negative Inotropic Activity^a^**	**Negative Chronotropic Activity^b^**	**Negative Inotropic Activity^c^**
**Curcuma extract**	27 ± 1.3	39 ± 2.1	33 ± 1.9
**Curcuma extract + atropine (1µM)**	21 ± 2.0	33 ± 3.1	30 ± 0.9
**Curcuma extract + DPCPX (1µM)**	25 ± 1.1	36 ± 1.8	34 ± 1.4

^a^ Activity: decrease in developed tension on isolated spontaneously beating mice atria at 1mg/ml, expressed as percent changes from the control (n = 4–6). ^b^ Activity: decrease on atrial rate in spontaneously beating isolated mice atria at 1 mg/ml, expressed as percent changes from the control (n = 6–8). Pretreatment heart rate ranged from 300 to 350 beats per min. ^c^ Activity: decrease in developed tension on isolated mice ventricula muscle driven at 1 Hz at 1mg/ml, expressed as percent changes from the control (n = 4–6).

The chronotropic and inotropic activities are commonly studied using the spontaneously beating right atrium and the electrically stimulated left atrium respectively, as a decrease of myocardial muscular contraction can be a consequence of a negative chronotropic effect [49]. 

However, due to the small dimensions of the mouse heart and the difficulty of separating the right from the left atrium, the inotropic effect was evaluated using the ventricles, driven at 1 Hz. In this experimental model the curcuma extract exerted also a weak negative inotropic effect (33 ± 1.9).

- *Aorta*

Curcuma extract (1 mg/ml) induced a transient (30 min) weak contraction (9 %) of mouse aorta muscular layer. Moreover, curcuma extract reduced 7 % potassium (80 mM) induced contraction and by 48 % noradrenaline (1 µM) mediated contractions.

- *Gastric fundus, bladder and trachea*

**Figure 5:**
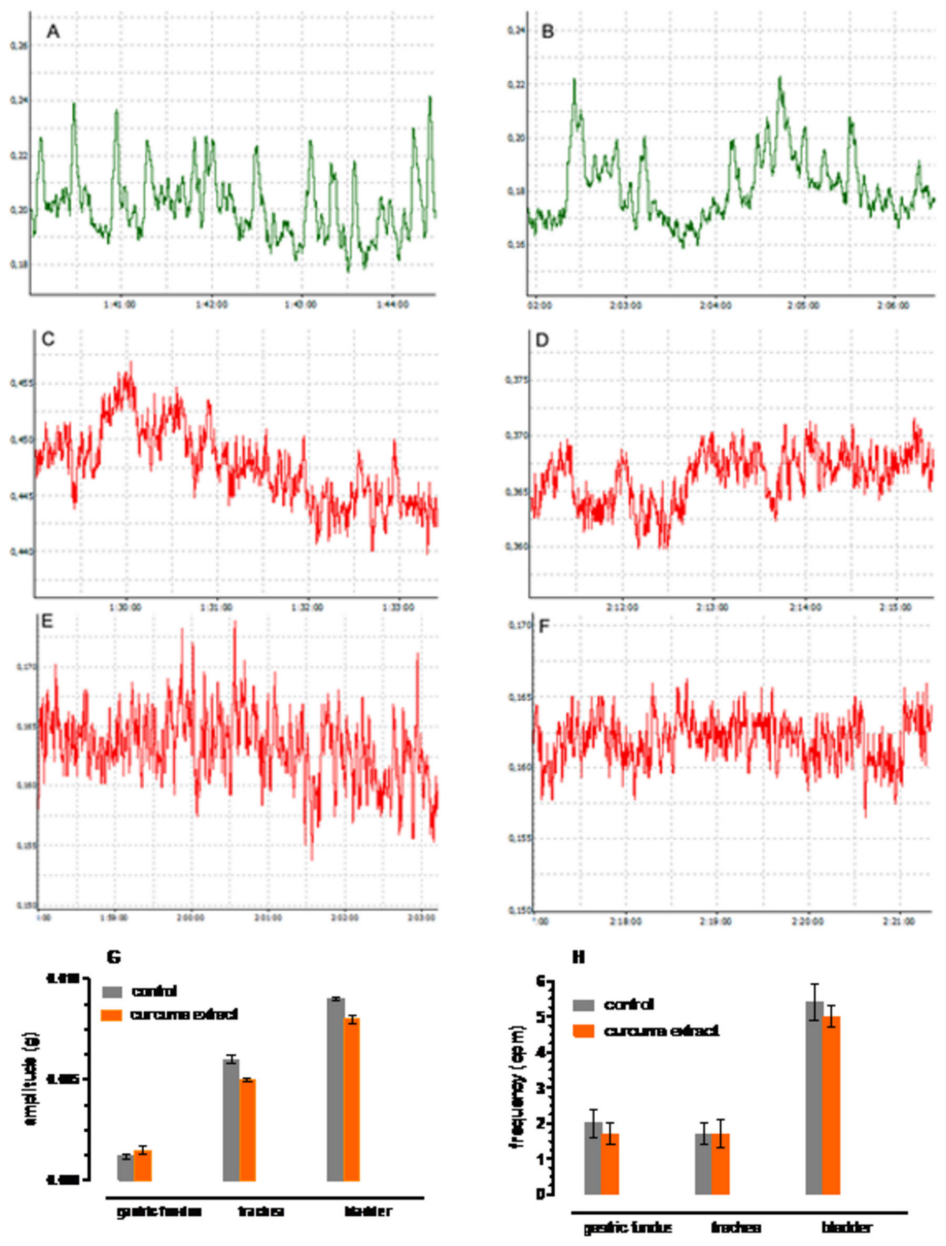
Basal contractile activity: effect of Curcuma extract 1 mg/ml. (A,B) baseline gastric smooth muscle phasic contractions before (A) and after curcuma extract addition (B). (C,D): races of baseline trachea smooth muscle phasic contractions before (C) and after curcuma administration (D ). (E,F): traces of baseline smooth muscle bladder phasic contractions before (E) and after curcuma administration (F). X axis (g) and Y axis (h). (G): spontaneous phasic contractions frequency in gastric fundus, trachea, bladder strips (mean ± SEM) (C): Spontaneous phasic contractions amplitude in gastric fundus, trachea, bladder strips (mean ± SEM). In all cases Curcuma vs controls *P* > 0.05.

Gastric fundus and bladder: Curcuma extract (1 mg/ml) had a dual effect: it did not modify the amplitude and frequency of spontaneous basal contractions of the smooth muscle strips (Figure 5A,B and Figure 5E,F respectively),. The frequencies of spontaneous phasic contractions of gastric fundus and bladder muscle strips from control and after addition of curcuma extract (1 mg/ml) were not significantly different (n = 5, *P* > 0.05) (Figure 5G). Similarly, the amplitudes of spontaneous phasic contractions of gastric fundus and bladder muscle strips were not significantly different from hose treated with curcuma extract (1 mg/ml) (n = 7, *P* > 0.05 (Figure 5H). Surprisingly curcuma extract (1 mg/ml) induced a stable contraction, without a modification if the tone, that was 23 % and 25 % of the Carbachol (1 µM) as reference in the stomach and bladder respectively. The contraction was reversed by 30 min washout; no effect was observed at 0.05 mg/ml Curcuma extract concentration. Trachea: The amplitude and the frequency of the spontaneous contractions of muscular strips after Curcuma extract addition (1mg/ml) were not significantly different from amplitude and frequency of control tissues (n = 6, *P* > 0.05) (Figure 5G and Figure 5H respectively) and induces a 24% relaxation on the tracheal rings, reversed by 30 min washing; no effect was observed at 0.5 mg/ml concentration. This effect is not mediated by the cholinergic system, since CCh induces contraction. 

- *Serum liver enzymes, lipids and glucose levels*

Curcumin administration did not result in any significant treatment-related change in clinical observations, body weights, body weight gains, feed consumption, and organ weights. No alterations were observed on the serum chemistry parameters and terminal necropsy did not reveal any treatment-related gross or histopathology findings.

Serum AST, ALT, total Cholesterol, HDL Cholesterol, Tryglicerides and Glucose levels did not change after 28 days of of *Curcuma Longa* L administration, (1g/ kg of 4RF21 complete food, 200 mg/Kg b.w./day) indicating the safety of the natural substance for the liver, lipid and glucose homeostasis (Table 4).

**Table 4:** Serum AST, ALT, total Cholesterol, HDL Cholesterol, Tryglicerides and Glucose level before and after *Curcuma Longa* administration.

	**AST^a^**	**ALT^b^**	**Total Chol^c^**	**HDL Chol^c^**	**Tryglicerides^d^**	**Glucose^d^**
Before Curcuma extract*	90.6 ± 10.3	45.7 ± 5.1	80.0 ± 10.7	70.3 ± 10.3	185.2 ± 20.6	180.3 ± 25.5
After Curcuma extract*	95.1 ± 6.5	44.4 ± 6.4	79.6 ± 15.4	71.4 ± 20.2	185.9 ± 40.3	168.9 ± 29.0

^a^ AST = Aspartate transaminases (Units/ml). ^b^ ALT = Alanine transaminases(Units/ml). ^c^ Chol = Cholesterol (mg/100 ml). ^d^ Expressed as mg/100 ml. In all cases, there was no statistical significance(p s.). * *Curcuma longa* Linn (Indena Spa, Milan, Italy) extract at a final concentration of 1g/ kg of 4RF21 complete food (200 mg/Kg b.w/day) was administered over 28 days to healthy animals.

#### Chronic curcuma administration

- *Bile Acids.*

Chronic feeding with Curcumin at a dose of 200 mg/Kg b.w to mice (1g/ kg of 4RF21 complete food) affects the bile acid biodistribution: the total bile acids concentration significantly increases both in the liver tissue and serum while it slightly decreases in bile. Bile acids in plasma, liver and bile are reported in Table 5.

**Table 5:** Plasma, liver and biliary bile acids before and after curcuma administration.

	**Bile** (mmol/l)	**Liver** (nmol/g)	**Plasma** (nmol/l)
**Before curcuma administration^a^**	363 ± 78	230 ± 87	301 ± 68
**After curcuma administration^a^**	324 ± 77	627 ± 90	860 ± 97
***P value^b^***	> 0.05 (n.s.)	≤ 0.05	≤ 0.05

^a^ Curcuma was administered to mice at a dose of 200mg/Kg/day over 28 days (1g/ kg of 4RF21 complete food) (see Materials and Methods). ^b^ Calculated by two-tail significance test, with a significance level of α = 0.05%. P≥ n.s.: not significant

However, after administration of curcuma, a variation of the endogenous bile acids composition was not observed compared to control sample pool.

## Discussion

In a previous paper we have suggested the use of *Curcuma Longa* extract as a therapeutical tool in diarrhea, due to its myorelaxant effect towards the intestinal muscle. Irritable bowel disease (IBS) is a worldwide diffused multifactorial pathophysiologal entity with multiple brain-gut and neuroimmune interactions [50]. A drug modulating gut motility can be a useful symptomatic tool. Since curcuma phytosomized extract absorption is 30 times higher than the common *Curcuma Longa* pharmacological available preparation, and its recovery therefore in the different organs is much higher, like also its biological activity towards several targets. In this view, it is worthwhile evaluating the safety profile and the effects of the preparation on the smooth muscle of different intestinal and extraintestinal districts, besides getting more insight into the mechanism of action of this substance in the gastrointestinal tract. The common used antidiarrheal drugs (5-HT_3-4_ receptor antagonists, lubiprostone, μ-opioid receptors agonist loperamide) [50] usually are hampered by side effects. Some herbal preparations are also used as a therapeutical means in irritable bowel disease, both in the constipation and in the diarrhea feature [51]. The so-called "natural drugs" used in alternative medicine can have dangerous adverse effects and the scientific field of activity associated with drug safety is increasingly becoming a major concern for the scientific community. In this study we focused mainly on the curcuma extract towards the neuronal and hormonal regulation of the smooth muscle layers in different organs, based on our previous observation showing a myorelaxant effect of *Curcuma Longa* L. on the mouse ileum and colon: The observed effect was due to a noncompetitive and transient inhibition of muscarinic receptors. In the present investigation we have also studied the effects of Curcuma extract on the autonomous system in different organs. In the intestine Curcuma activity involves not only the cholinergic receptors, but also L type Calcium channels and the effect is stronger in the ileum than in the colon. It is noteworthy that curcuma extract also completely inhibits histamine contraction. The latter finding justifies the potential clinical use of curcuma extract in patients affected by IBS, who have an increase of pro-inflammatory mediators and present an augment of mast cells in close apposition to nerves, determining colorectal hypersensitivity [52]. The same observation holds for the effect on 5-HT receptors: In fact, 5-HT plays a key role in the control of gastrointestinal motility, secretion and sensitivity [53] and its spontaneous release is significantly increased in IBS patients and it may contribute to abdominal pain [54]. 5-HT_3_ serotonin antagonists used for diarrhea are not devoid of side effects: for example, alosetron has been suspected to determine colonic ischemia [55]. In the present experimental model, serotonin induces a transient contraction of the smooth muscle, mediated by cholinergic receptors, followed by a relaxation, mediated by a direct agonism towards serotoninergic receptors. Curcuma extract abolishes the contractile initial response through its antagonism against cholinergic system, at the dose representing the IC_50_ for the colon, therefore proving an antiserotoninergic effect. These results support the use of curcuma extract as a possible tool in the management of gastrointestinal tract dysmotility disorders. Since antidiarrheal drugs [56,57] decrease gallbladder motility, impairing gallbladder emptying, their chronic administrations may contribute to gallstone formation [58]. Basing on this observation, we argued whether curcuma extract administration contribute to cholelithiasis. Curcuma extract, at the dose effective on the intestinal tract contracts gallbladder smooth muscle: this result is consistent with the data obtained by Rasyid and colleagues [59], who observed that curcuma induces contraction of the human gallbladder, thus ruling out a possible side effect of this natural substance. In addition, curcuma extract does not inhibit gastric motility even at a high concentration: on the opposite it induces a contraction of the fundus that is 23% of the Carbachol induced contraction; the concentration effective on the ileum does not induce any effect on the mouse stomach. The same mild contraction was also observed in the urinary bladder and trachea and it was demonstrated to be reversible and independent of the cholinergic receptors, at a dose 300-400 times higher than the IC_50_ effective dose in the intestine. In addition, this contraction does not occur at the dose representing the IC_50_ for the colon therefore ruling out any possible antitarget effect of producing dysuria and bronchospasm. The same observations hold for the great vessels: curcuma extract induced a weak contraction of the aorta at the same high dose and it has a minimal effect towards L type calcium channels in the muscular layers of this vessel. This finding is at variance with the high selectivity of Curcuma with the LTCC in the present in the smooth muscle of the ileum and colon, where the K^+^-induced contraction is reduced by 100% and 84% respectively. In addition, the negative inotropic and chronotropic effect of Curcuma on heart contractility and rate is present at a dose by far higher than the IC_50_ effective dose on the ileal and colonic muscular layers.

However, although in presence of normal liver parameters and serum lipids and glucose, it is worthwhile considering that both plasma and liver bile acid concentration is increased: this latter finding is in agreement with the data obtained by Deters and coworkers [40], who have described that chronic Curcumin administration (100 mg/kg b.w./day) induces a slight decrease of bile flow (-7 %) and biliary bile acid secretion in bile fistula rat (-12 %), without effect on biliary secretion of cholesterol. The dose we have used in the present investigation is twice the dose used by Deters and colleagues [40] and it is possible that the effect observed in the present investigation is dose dependent. These data suggest that administration of curcuma, at least in rodents determines a mild cholestatic effect, responsible for an accumulation of BA in the liver cell and an increased back diffusion to the systemic compartment. The 2-3 fold increased bile acid serum concentration is still in the physiological range, similarly to the increased serum concentration that occur after feeding with respect to fasting conditions. Curcuma extract stimulates gallbladder motility and increased bile acids serum levels could result from a sustained BA biliary secretion following gallbladder emptying with a continuous input of the BA to the liver via portal vein. However, this can hardly explain the increased BA concentration in the liver. Biliary excretion of curcumin is mediated by multidrug resistance-associated protein 2 (Mrp1, *Abcc1*) [60] and Curcumin has been demonstrated to inhibit both Mrp1 and multidrug resistance-associated protein 2 (Mrp2, *Abcc2*)-mediated transport [61]. No information is available about any inhibition/ competition between curcuma extract and bile acids in the rat for Bile Salt Export Pump (Bsep, *Abcb11*), the ATP-dependent export system of bile acids [62]. Since only bile salts bearing two negative charges, such as sulphated tauro- or glycoconjugated bile acids share Mrp2 transport with curcumin and sulphated BA conjugates are mostly present in cholestasis, it is difficult to explain the high liver and plasma concentration of bile acids after curcuma extract chronic feeding in mouse, by a competition of di-anionic conjugated bile acids and curcuma extract for the Mrp2 transport system [63,64]. 

The different biodistribution of bile acids in the plasma-liver-bile compartment deserves further investigation, mainly in view of a chronic administration of this natural substance. The present investigation supports the concept of the importance of the evaluation of the antitarget effects also of natural substances that are largely used as a self medication and in folk medicine, in order to provide a pharmacological safety profile.
